# EFFECTS OF PRODUCTIVE ENGAGEMENT ON SUBJECTIVE HEALTH AMONG OLDER ADULTS WITH SENSORY IMPAIRMENTS

**DOI:** 10.1093/geroni/igz038.3058

**Published:** 2019-11-08

**Authors:** Othelia E Lee, Junghyun Park

**Affiliations:** 1 University of North Carolina Charlotte, Charlotte, United States; 2 New York University, NY, New York, United States

## Abstract

Background: Productive engagement becomes significant protective factors in healthy aging. Yet, subgroups of older adults with age-related vision and hearing impairments lack access to various activities , suggesting that unequal ability to participate in productive aging is a major public health and health-disparities concern. Methods: Older adults experiencing age-related vision and hearing impairments were drawn from the 2015-2017 National Survey on Drug Use and Health (n=2,164). Perceived health status (good vs. poor) was outcome measures used in multivariate logistic regression. Two aspects of productive engagement was considered: 1) employment status (unemployed vs employed) and 2) regular religious service attendance as tools to build social capital in their faith-based communities. Gender, race, marital status, educational attainment, poverty, urbanization, obesity, chronic disease, hospitalization, binge drinking, cigarette smoking, and difficulty with mobility were considered as covariates. Results: Working older adults with sensory loss were more likely to perceived good health status, compared to their unemployed counterparts (OR=2.46, p<.05). Religious service attendance also became protective factors for health (OR=1.60, p<.01). Of the covariates, higher educational attainment, White race, having one chronic disease, hospitalization, smoking, drinking, and mobility challenges appeared to affect the health status. Conclusions/Implications: Study findings implied the needs to identify late-life engagement through work and participation in faith-based community as a major public health issue. Given the barriers and disincentives to the productive engagement of older adults in this culture, healthcare providers should provide programs promoting employment and religious attendance.

